# MDM2-Driven Ubiquitination Rapidly Removes p53 from Its Cognate Promoters

**DOI:** 10.3390/biom12010022

**Published:** 2021-12-24

**Authors:** Kester Mo Henningsen, Valentina Manzini, Anna Magerhans, Sabrina Gerber, Matthias Dobbelstein

**Affiliations:** Institute of Molecular Oncology, Göttingen Center of Molecular Biosciences (GZMB), University Medical Center Göttingen, D-37077 Göttingen, Germany; k.henningsen@stud.uni-goettingen.de (K.M.H.); vmanzin@gwdg.de (V.M.); anna.magerhans@med.uni-goettingen.de (A.M.); sabrina.gerber@med.uni-goettingen.de (S.G.)

**Keywords:** p53, MDM2, ubiquitination, DNA binding, MDMX, Nutlin

## Abstract

MDM2 is the principal antagonist of the tumor suppressor p53. p53 binds to its cognate DNA element within promoters and activates the transcription of adjacent genes. These target genes include MDM2. Upon induction by p53, the MDM2 protein binds and ubiquitinates p53, triggering its proteasomal degradation and providing negative feedback. This raises the question whether MDM2 can also remove p53 from its target promoters, and whether this also involves ubiquitination. In the present paper, we employ the MDM2-targeted small molecule Nutlin-3a (Nutlin) to disrupt the interaction of MDM2 and p53 in three different cancer cell lines: SJSA-1 (osteosarcoma), 93T449 (liposarcoma; both carrying amplified MDM2), and MCF7 (breast adenocarcinoma). Remarkably, removing Nutlin from the culture medium for less than five minutes not only triggered p53 ubiquitination, but also dissociated most p53 from its chromatin binding sites, as revealed by chromatin immunoprecipitation. This also resulted in reduced p53-responsive transcription, and it occurred much earlier than the degradation of p53 by the proteasome, arguing that MDM2 removes p53 from promoters prior to and thus independent of degradation. Accordingly, the short-term pharmacological inhibition of the proteasome did not alter the removal of p53 from promoters by Nutlin washout. However, when the proteasome inhibitor was applied for several hours, depleting non-conjugated ubiquitin prior to eliminating Nutlin, this compromised the removal of DNA-bound p53, as did an E1 ubiquitin ligase inhibitor. This suggests that the ubiquitination of p53 by MDM2 is necessary for its clearance from promoters. Depleting the MDM2 cofactor MDM4 in SJSA cells did not alter the velocity by that p53 was removed from promoters upon Nutlin washout. We conclude that MDM2 antagonizes p53 not only by covering its transactivation domain and by destabilization, but also by the rapid, ubiquitin-dependent termination of p53–chromatin interactions.

## 1. Introduction

The MDM2 oncoprotein is the major antagonist of the tumor suppressor p53 [1,2,3]. The p53 protein consists of an amino-terminal transactivation domain, a central DNA binding domain and a carboxyterminal homotetramerization domain. The p53 tetramer binds to the DNA of promoters and activates the transcription of the adjacent genes. The MDM2 gene is one of these p53-inducible genes, thereby forming a negative regulatory feedback loop. When MDM2 levels are enhanced, MDM2 physically interacts with p53, mainly through the association of the amino–terminal transactivation domain of p53 with the amino–terminal hydrophobic pocket domain of MDM2. This interaction results in p53 ubiquitination, mediated by the carboxyterminal RING finger domain of MDM2 through its E3 ubiquitin ligase activity. The interaction is amenable to disruption by small molecules that occupy the p53-binding site within MDM2 by covering the hydrophobic pocket. The prototype of such drug candidates consists in Nutlin-3a [4], hereafter termed Nutlin.

The MDM4 (alias MDMX) protein structurally resembles MDM2 and contains a RING finger domain, as well [3]. The MDM4 RING finger does not display any known E3 ligase activity on its own, but it associates with MDM2 to fortify its activity to antagonize p53 [5]. Moreover, the targeted disruption of either MDM2 or MDM4 gives rise to p53-dependent embryonic lethality [6], making it clear that both are needed for balancing p53 under physiological circumstances. Biochemically, MDM2 on its own was found to transfer a limited number of ubiquitin moieties on p53 in vitro, whereas the combination of MDM2 and MDM4 are polyubiquitinating p53 [7].

When p53 is induced, e.g., by DNA damage, MDM2 typically accumulates with a delay, presumably as a means to terminate p53 activity when damage is repaired. The adjustment to the initial levels of p53 typically takes at least several hours [8]. This leaves two open questions. Firstly, is there a fast way to terminate p53 activity when needed, e.g., to avoid unnecessary apoptosis? And secondly, how is the turnover of DNA-bound p53 regulated by the proteasome? Considering that most active proteasomes reside in the cytoplasm [9] but not on chromatin, especially in proliferating cells [10], p53 first had to dissociate from its cognate DNA binding sites before being degraded. If this had to occur spontaneously, it can become a limiting factor for antagonizing p53. We therefore asked if MDM2 can confer mechanisms that dissociate p53 from chromatin.

Some previous studies actually suggest that MDM2 can loosen the interaction of p53 with its cognate promoters, although some of the results seem contradictory and/or were only obtained in vitro, rather than in cells. On the one hand, MDM2 was found to induce a mutant-like conformation of p53 that precludes DNA binding [11]. On the other hand, the ubiquitination of p53 (at least when artificially fusing p53 to ubiquitin) was found to diminish its association with DNA [12] and enhance its nuclear export [13]. Furthermore, MDM2 was reported to associate with DNA-bound p53 and form a repressive complex [14], perhaps by recruiting histone deacetylases (HDACs) [15]. Thus, it remains elusive whether MDM2 not only covers the transactivation domain of p53 but also loosens the association of p53 with promoter DNA in vivo, and especially whether MDM2 removes activated and prebound p53 from promoters to terminate its activity. If so, the additional question remains whether this removal of p53 merely requires the association of MDM2 with p53, or whether it also involves ubiquitination. Finally, the role of MDM4 in such a process requires clarification.

In the present paper, we show that MDM2 can remove p53 from promoters within minutes after the two proteins are allowed to associate, in parallel to fast p53 ubiquitination, but much earlier than the degradation of p53. The dissociation of p53 from promoters does not require proteasomal activity but does rely on available ubiquitin. MDM4, while contributing to the polyubiquitination of p53, is not required for the removal of p53 from promoters, arguing that MDM2-driven ubiquitination is the key mechanism for the fast elimination of p53 from its cognate promoter elements.

## 2. Materials and Methods

### 2.1. Cell Culture

SJSA cells, derived from osteosarcoma, MCF7 cells, derived from mammary adenocarcinoma, and the liposarcoma-derived 93T449 cells were cultured in Dulbecco’s modified Eagle’s medium (DMEM) with additional 10% fetal bovine serum (FBS), 2 mM glutamine, 50 μg/mL streptomycin, 50 U/mL penicillin, and 2 µg/mL tetracycline. The cells were tested regularly to exclude contaminations with mycoplasmas.

### 2.2. Treatments

The cells were treated with the following compounds: 20 µM Nutlin-3a (Nutlin; Cat. #: B0084-425358, BOC Sciences, Shirley, NY, USA); 20 µM MG-132 (Cat. #: 474791, Millipore, Billerica, MA, USA); 20 µM PYR-41 (Cat. #: 662105, Thermo Fisher Scientific, Waltham, MA, USA); 250 µg/mL neocarzinostatin (Cat. #: N9162 Sigma-Aldrich, St. Louis, MO, USA); and dimethyl sulfoxide (DMSO, Cat. #: A3672,0100, AppliChem, Darmstadt, Germany).

For Nutlin washout, SJSA were treated with 20 µM Nutlin-3a for 4 h and Nutlin was washed out by removing the Nutlin-containing medium, washing once with PBS and adding the fresh medium without Nutlin.

### 2.3. Transfections

Cells were reverse (i.e., in suspension) transfected using lipofectamine 3000 (Cat. #: 11668-019, Invitrogen, Carlsbad, CA, USA) with 10 nM siRNA against MDM2 (s8629, Cat. #: 4392420, Thermo Fisher Scientific), MDM4 (s8632, Cat. #: 4392420, Thermo Fisher Scientific), or negative scrambled control siRNA (Cat. #: 4390847, Thermo Fisher Scientific). The cells were incubated for 48 h prior to harvest, but the medium was refreshed after 24 h.

### 2.4. Immunoblot Analysis

Cells were washed with cold PBS once and harvested on an ice in protein lysis buffer, i.e., 20 mM Tris-HCl pH 7.5; 150 mM NaCl; 10 mM EDTA; 1% Triton-X 100; 1% deoxycholate salt; 0.1% SDS; 2 M urea; and protease inhibitors (pepstatin, leupeptin hemisulfate, aprotinin, AppliChem). For the disruption of chromatin, the samples were briefly pushed through a syringe (Cat. #: 4657675, Braun, Kronberg im Taunus, Germany) and sonicated with a Bioruptor (Diagenode, Denville, NJ, USA). A BCA protein assay kit (Cat. #: 23227, Thermo Fisher Scientific) was used for measuring the protein concentration. Accordingly, equal amounts of protein were boiled in Laemmli buffer at 95 °C for 5 min and separated by SDS-PAGE. The proteins were then transferred onto a 0.2 µm nitrocellulose membrane. The membranes were blocked for 1 h in TBS with 0.1% Tween-20 and 5% non-fat milk and incubated with the following primary antibodies at 4  °C overnight: p53 DO-1 (Cat. #: sc126, Santa Cruz, Dallas, TX, USA); MDM2 (IF2, Cat. #: OP46, Millipore); phospho-p53 (Ser20) (Cat. #: 9287T, Cell Signaling, Danvers, MA, USA); phospho-histone H2A.x (Ser139) (Cat. #: 9718S, Cell Signaling); p21 (Cat. #: 2947S, Cell Signaling); HIF-1 α(Cat. #: 3716S, Cell Signaling); β actin (Cat. #: ab6276, Abcam, Cambridge, UK); and GAPDH (Cat. #: ab8245, Abcam).

Subsequently, membranes were incubated with peroxidase-conjugated secondary antibodies, either donkey anti-rabbit IgG (Cat. #: 711-036-152, Jackson ImmunoResearch, Philadelphia, PA, USA) or donkey anti-mouse IgG (Cat. #: 715-036-150, Jackson ImmunoResearch), followed by detection using a Universal Hood III (Bio-Rad Laboratories, Hercules, CA, USA) and either Immobilion Western Substrate (Cat. #: WBKLS0500, Millipore) or Super Signal West Femto Maximum Sensitivity Substrate (Cat. #: 34095, Thermo Fisher Scientific).

### 2.5. Chromatin Immunopreciptitation (ChIP)

Chromatin immunoprecipitation was carried out as described by our group [16,17] but modified as follows. The cells were cultivated while adherent on 145 mm dishes (Cat. #: 639160, Greiner Bio-One, Frickenhausen, Germany), washed with cold PBS once and crosslinked for 40 min in PBS with 2 mM of the protein–protein crosslinker ethylene glycol-bis(succinimidylsuccinate) (EGS, Cat. #: 21565, Thermo Fisher Scientific), and then DNA–protein-crosslinked with 1.1% of paraformaldehyde (PFA) in PBS for 30 min on the dish. Fixation was quenched by adding 0.125 M glycine for 5 min. The cells were washed twice with cold PBS and incubated on the dish in 2 mL of lysis buffer, consisting of 20 mM HEPES, 10 mM EDTA, 0.5 mM EGTA and 0.25% Triton X-100 for 10 min at 6 °C to perforate the cell membrane, then carefully scraped off, transferred to tubes and incubated again for 10 min on ice. The nuclei were separated from the cytosol by centrifuging them for 10 min at 250× *g* and 4 °C, discarding the supernatant, washing once with 50 mM HEPES, 1 mM EDTA, 0.5 mM EGTA and 150 mM NaCl, centrifuging for 10 min at 250× *g* and 4 °C and discarding the supernatant again. The nuclei were resuspended in 20 mM HEPES, 0.15 M NaCl, 1 mM EDTA, 0.5 mM EGTA, 1% Triton X-100, 0.375% SDS and protease inhibitors (cOmplete Mini, Cat. #: 11836170001, Roche, Basel, Switzerland) to perforate the nuclear membrane. Samples were sonicated with a Bioruptor Pico (Diagenode) for 15 cycles (SJSA) or 20 cycles (MCF7, 93T449) with settings 30 sec on and 30 sec off per cycle in Bioruptor Microtubes (Cat. #: C3001006, Diagenode) to open the remaining nuclei and shear the chromatin. To adjust the amounts of chromatin for the immunoprecipitation, a fraction of the cell lysate (30 µL) was incubated with RNase A, de-crosslinked and purified to measure its concentration with a spectrophotometer (Nanodrop, ND-1000, Peqlab, Erlangen, Germany), and the remaining chromatin was diluted accordingly. A small amount of the diluted lysate was then preserved as input while the remaining lysate was incubated at 4 °C overnight with Protein A/G PLUS-Agarose (sc-2003, Santa Cruz) and 2 µg of the following antibodies: pre-immune IgG (Cat. #: 171870, Abcam); p53, carboxyterminal (Cat. #: C15410083, Diagenode); p53 PAb1801 (Cat. #: OP09, Millipore); p53 PAb421 (Cat. #: OP03, Millipore) and p53 DO-1 (Cat. #: sc126, Santa Cruz). After washing the beads 6 times, the input and beads were incubated at 37 °C for 30 min in 10 mM Tris pH 8.0 and 0.2 µg/µL RNase A (Cat. #: 1007885, Qiagen, Hilden, Germany). The DNA–protein complexes were then de-crosslinked by adding 50 µL 100 mM Tris pH 8.0, 20 mM EDTA, 2% SDS and 0.4 µg/µL proteinase K (Cat. #: 25530-049, Invitrogen), and incubating in a thermocycler (Eppendorf, Hamburg, Germany) with 800 rpm at 65 °C overnight. The DNA was purified with a MinElute PCR Purification Kit (Cat. #: 28006, Qiagen) and quantified with targeted qPCR.

### 2.6. Co-Immunoprecipitation (Co-IP)

SJSA cells were washed with cold PBS followed by lysis with a Co-IP buffer consisting of 50 mM Tris-HCl pH 7.5, 300 mM NaCl, 1% NP40 and protease inhibitors (cOmplete Mini, Cat. #: 11836170001, Roche). The lysate was then homogenized and sonicated as described for the immunoblot analysis and precleared by incubation for 2 h at 4 °C with Protein G Sepharose (PGS, GE Healthcare, Chicago, IL, USA). The precleared lysate was incubated at 4 °C overnight with 3 µg of antibodies, p53, carboxyterminal (Cat. #: C15410083, Diagenode); p53 PAb1801 (Cat. #: OP09, Millipore); p53 PAb421 (Cat. #: OP03, Millipore); p53 DO-1 (Cat. #: sc126, Santa Cruz) or β-Galactosidase (Cat. #: Z378B, Promega, Madison, WI, USA). Afterwards, the protein–antibody complex was coupled to the PGS by incubation for 2 h at 4 °C, and the beads were then washed 3 times with a Co-IP buffer, resuspended in a Laemmli buffer, and boiled at 95 °C for 5 min prior to separation by SDS-PAGE. The transfer to nitrocellulose and signal detection was performed as described above, except that an anti-p53 antibody (p53 DO-1 HRP, Cat. #: sc-126 HRP, Santa Cruz) or anti-ubiquitin antibody (Ubiquitin PD41 HRP, Cat. #: sc-8017 HRP, Santa Cruz) covalently conjugated to horseradish peroxidase was used for detection.

### 2.7. RNA Isolation and cDNA Synthesis

The SJSA cells were washed with cold PBS once and then lysed in 1 mL of phenol containing TRIzol reagent (Cat. #: 15596018, Thermo Fisher Scientific). For RNA isolation, 200 µL of chloroform was added, and the samples were centrifuged for phase separation. The aqueous phase containing the RNA was isolated, and RNA was precipitated by 20 min incubation at room temperature in 500 µL isopropanol. After washing with 75% ethanol, air drying and resuspension in nuclease-free water, the RNA amount was measured by spectrophotometry (Nanodrop, ND-1000, Peqlab) and diluted accordingly. Because of our interest in nascent RNA, we performed DNase treatment by 30 min incubating 2.2 µg of RNA at 37 °C with 2 U DNase I, RNase-free (Cat. #: EN0521, Thermo Fisher Scientific), 20 U RNase inhibitor (Cat. #: EN0381, Thermo Fisher Scientific) and DNase reaction buffer with MgCl_2_ (Cat. #: B43 Thermo Fisher Scientific). DNase was inactivated by the addition of 5 mM EDTA for 10 min at 65 °C. The RNA was used as a template for cDNA synthesis. A total of 6.25 µM of oligo-dT and 1.875 µM of random nonanucleotide primers (Metabion, Planegg, Germany), as well as 0.625 mM dNTPs (dATP, dCTP, dGTP, dTTP, Cat. #: 1202, 1203, 1204, 1205, Primetech, Minsk, Belarus), was added to 1 µg of RNA, and it was incubated for 5 min at 70 °C for RNA denaturation. For cDNA synthesis, a transcriptase buffer (Cat. #: B0253S, New England BioLabs, Ipswich, MA, USA), 10 U of RNase inhibitor, human placenta (Cat. #: M0307L, New England BioLabs) and 25 U of M-MuLV reverse transcriptase (Cat. #: M0253S, New England BioLabs) was added to each sample, and it was incubated for 1 h at 42 °C, followed by 5 min incubation at 95 °C for inactivating the reverse transcriptase. A control reaction to the cDNA synthesis was performed, omitting the reverse transcriptase (no reverse transcriptase controls, NRTs), to exclude the DNA contamination of the RNA samples. The cDNA was diluted with ddH_2_O and stored for qPCR. Each biological replicate of ChIP or RNA analysis consisted of technical duplicates or triplicates in the qPCR.

### 2.8. Real-Time Quantitative Polymerase Chain Reaction (qPCR)

The quantification of DNA from ChIP or cDNA from RNA isolation was performed by a real-time qPCR, performed in a thermocycler (CFX96 Bio-Rad Laboratories). For each reaction, 25 µL of reaction mix was put into 96-well plates.

The reaction mixture for cDNA was 6 µL of ddH_2_O, 3 µL of cDNA template, 1 µL of 10 µM forward and the same amount of reverse primer, as well as 14 µL of a homemade qPCR mixture. The qPCR mixture contained 535 mM trehalose; 75 mM Tris-HCl pH 8.8; 20 mM ammonium sulphate; 0.01% Tween-20; 0.25% Triton X-100; 3 mM MgCl_2_, 0.45 concentrated SYBR Green I (from a 10,000× stock solution) (Cat. #: S7567, Thermo Fisher Scientific); 0.2 mM dNTPs (dATP, dCTP, dGTP, dTTP, Cat. #: 1202, 1203, 1204, 1205, Primetech) and 20 U/mL Taq-Polymerase (Cat. #: 1800, Primetech). The program used for the thermocycler was 2 min at 95 °C followed by 45× cycles of 15 s at 95 °C and 1 min at 60 °C. Subsequently, a melting curve was generated, followed by a final incubation for 30 s at 95 °C.

For DNA from ChIP, 5 µL of template DNA was mixed with 1 µL of 10 µM forward primer and the same amount of reverse primer, 5.5 µL of ddH_2_O and 12.5 µL of Maxima SYBR Green qPCR Master Mix (2×) (Cat. #: K0253, Thermo Fisher Scientific). The program used for the thermocycler was 10 min at 95 °C followed by 45× cycles of 15 s at 95 °C and 1 min at 60 °C, a melting curve generation and 30 s at 95 °C.

The primer sequences were as follows (Table 1 and Table 2).

### 2.9. Statistical Analysis and Software

The quantification of immunoblot analyses was performed using the volume tool of Image Lab 5.2 (Image Lab Software, Bio-Rad Laboratories). A rectangle was drawn around the bands of interest, and the mean value of intensity inside the rectangle was quantified. To assure the comparability of the lanes, rectangles covered the same area size. All intensities were quantified relative to the intensities of the associated loading controls. Exemplary rectangles are shown in Supplementary Figure S5A.

The quantification of the qPCR signal was performed according to the comparative threshold (2^−ΔΔCt^) method, relative to the chromatin input (for ChIP) or to the housekeeping gene 36B4 (for RNA and NRT control measurement). For the comparison of biological replicates, the enrichment relative to the input or the housekeeping gene was then normalized to a control sample, as stated in the figure legends for each experiment.

Statistical testing was performed using GraphPad Prism 8 (GraphPad Software, San Diego, CA, USA) or Excel. For the calculation of the significance, 2-sided unpaired Student’s t-tests were used.

## 3. Results

### 3.1. P53 Is Rapidly Removed from Promoters upon Nutlin Washout, in Parallel to Its Ubiquitination, but Prior to Its Degradation

**Figure 1:**
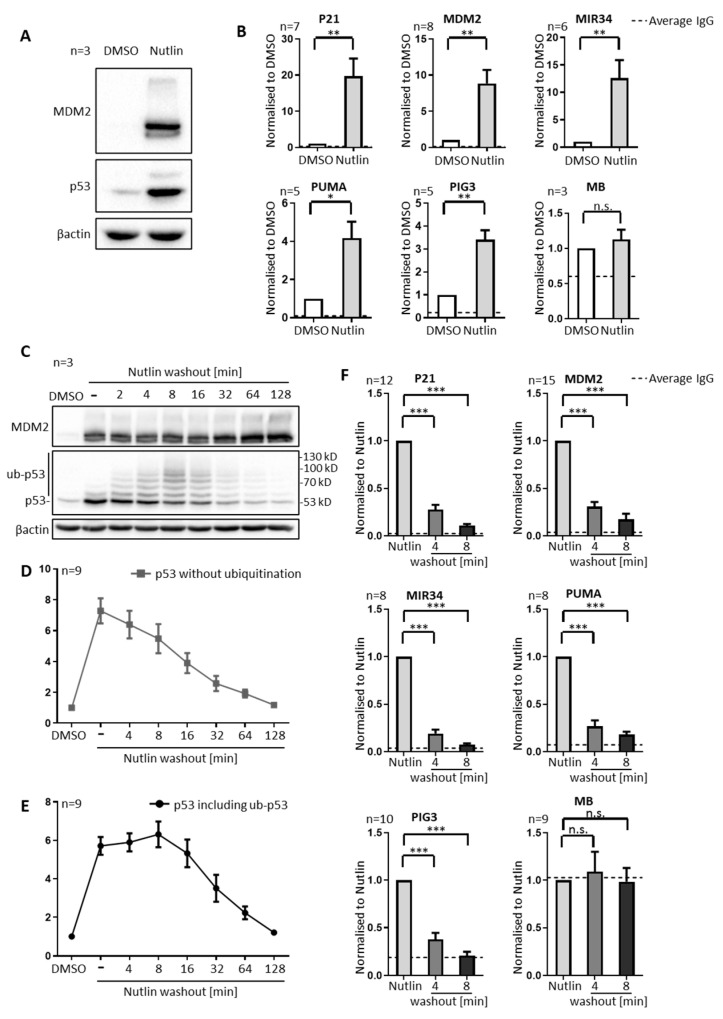
Immediate elimination of p53 from promoter DNA upon Nutlin washout. Experiments were performed in SJSA cells treated with 20 µM of Nutlin, or the amount of the DMSO solvent alone, for 4 h. (**A**) A representative immunoblot (out of three) showing the induction of p53 and MDM2 in SJSA cells upon Nutlin treatment. (**B**) Chromatin immunoprecipitation (ChIP) analyses followed by quantitative real-time PCR (qPCR) were performed to determine the degree that p53 was occupying the promoters of its target genes P21 (CDKN1A), MDM2, MIR34 (MIR34AHG), PUMA (BBC3), PIG3 (TP53I3) and myoglobin (MB, negative control). The analysis revealed a significant increase in chromatin-bound p53 upon Nutlin treatment at target genes but not at the negative control site. Enrichment of chromatin-bound p53 was normalized to the amount of chromatin input and to the DMSO-treated sample and displayed as mean + SEM from the indicated number (n) of biological replicates. The dotted line shows the background signal, i.e., the average enrichment upon chromatin immunoprecipitation with unspecific immunoglobulin G (IgG). (**C**) P53 is progressively ubiquitinated and degraded upon Nutlin treatment and subsequent washout, while MDM2 remains stable throughout the observation period of 128 min (immunoblot analysis). (**D**) Quantification of the signal corresponding to non-modified p53, from immunoblot analyses of nine biological replicates, including the ones that are shown in (**C**) and in Figure 3 through 8. Results are displayed as mean + SEM. (**E**) Analysis equivalent to (**D**) but now comprising both non-modified p53 and ubiquitinated p53 with higher apparent molecular weight, reflecting early ubiquitination but slower degradation. (**F**) ChIP analysis performed as described in (**B**) but comparing the association of p53 with promoters before and shortly after Nutlin washout. Of note, p53 was found removed from its target gene promoters within four and eight minutes of Nutlin washout. *** *p* < 0.001; ** *p* < 0.01; * *p* < 0.05; n.s., non-significant.

To assess the degree of ubiquitination and chromatin binding of p53, we treated SJSA cells transiently with Nutlin. SJSA cells are derived from an osteosarcoma; they contain wildtype p53, and the MDM2 gene in amplified copy numbers, resulting in high MDM2 levels to neutralize p53. As expected, Nutlin increased the levels of p53 (for lack of MDM2-mediated ubiquitination) and MDM2 (since MDM2-expression is driven by p53), as revealed by immunoblot analysis (Figure 1A). Applying Nutlin for different time periods gradually increased the levels of p53 and (with some delay) MDM2 (Supplementary Figure S5B), similar to previous observations [8]. In parallel, the amount of promoter-bound p53 was strongly enhanced by Nutlin, as determined by the chromatin immunoprecipitation (ChIP) and quantitative PCR-amplification of p53-responsive promoter elements (Figure 1B). P53 occupancy was detected at the promoters of its target genes P21 (CDKN1A), MDM2, MIR34 (MIR34AHG), PUMA (BBC3) and PIG3 (TP53I3). A portion of the myoglobin gene (MB) served as a negative control. Nutlin had no effect on the p53 occupancy at the myoglobin gene, nor was p53 ChIP enriched in comparison to an unspecific IgG antibody at this gene.

Next, we replaced the Nutlin-containing culture medium with fresh medium that did not contain drugs (further referred to as Nutlin washout). Remarkably, it took less than five minutes to start the accumulation of high molecular weight forms of p53 (Figure 1C), at least compatible with the accumulation of ubiquitinated p53, and in agreement with previous in vitro and in vivo studies [18,19], in which this pattern was shown to represent ubiquitinated p53. For additional confirmation, we immunoprecipitated p53, followed by immunoblot analysis detecting ubiquitin. Here, again, we observed the accumulation of high molecular proteins upon Nutlin washout (Supplementary Figure S5C). 

Even more strikingly, the association of p53 with the promoter-DNA of its target genes was diminished within the same short time frame. We observed that, in SJSA cells, more than half of the promoter occupancy by p53 at its target genes (but not at the myoglobin gene) was abolished within four minutes after Nutlin was washed out, and that the majority of p53 was removed within eight minutes (Figure 1F). In the MCF7 and 93T449 cells, too, we observed the ubiquitination of p53 by immunoblot analysis, and we also found the rapid removal of p53 from its target gene promoters upon Nutlin by chromatin immunoprecipitation (Supplementary Figure S1A,C). The removal was particularly fast in SJSA and 93T449 cells, which both carry an amplified MDM2 gene, and it was somewhat slower in MCF7 cells, which contain an amplification of MDM4 but not MDM2 [20]. This suggests that the velocity of removing p53 from its cognate promoters depends on the levels of MDM2.

To exclude epitope-masking by MDM2 as a possible confounder, we recapitulated the experiment suing four different antibodies that target p53 at different epitopes (Supplementary Figure S4). While the overall efficiency of chromatin immunoprecipitation differed between these antibodies, the rapid removal of p53 was detectable by all of them. Furthermore, we performed immunoprecipitaion with all antibodies, showing that they precipitate ubiquitinated p53 upon Nutlin washout (Supplementary Figure S5C).

By quantifying the band intensities of non-modified p53 in SJSA cells on immunoblots, we detected a relevant decrease only after p53 had dissociated from the promoters (Figure 1D). The loss in overall p53, determined by summing up the band intensities corresponding to non-modified as well as ubiquitinated p53, only became obvious at ca. 30 min after Nutlin washout, i.e., far later than ubiquitination and the loss of promoter binding (Figure 1E). In fact, while non-ubiquitinated p53 quickly began to diminish, there was a plateau in the overall p53 levels within the first eight minutes after Nutlin washout, indicating that p53 was mainly ubiquitinated but not degraded within this early time frame. Taken together, these results provide a first hint that the removal of p53 from promoters upon MDM2 activation does not require p53 degradation, but can involve ubiquitination.

### 3.2. Nutlin Washout Diminishes p53-Mediated Transcription within an Hour

**Figure 2:**
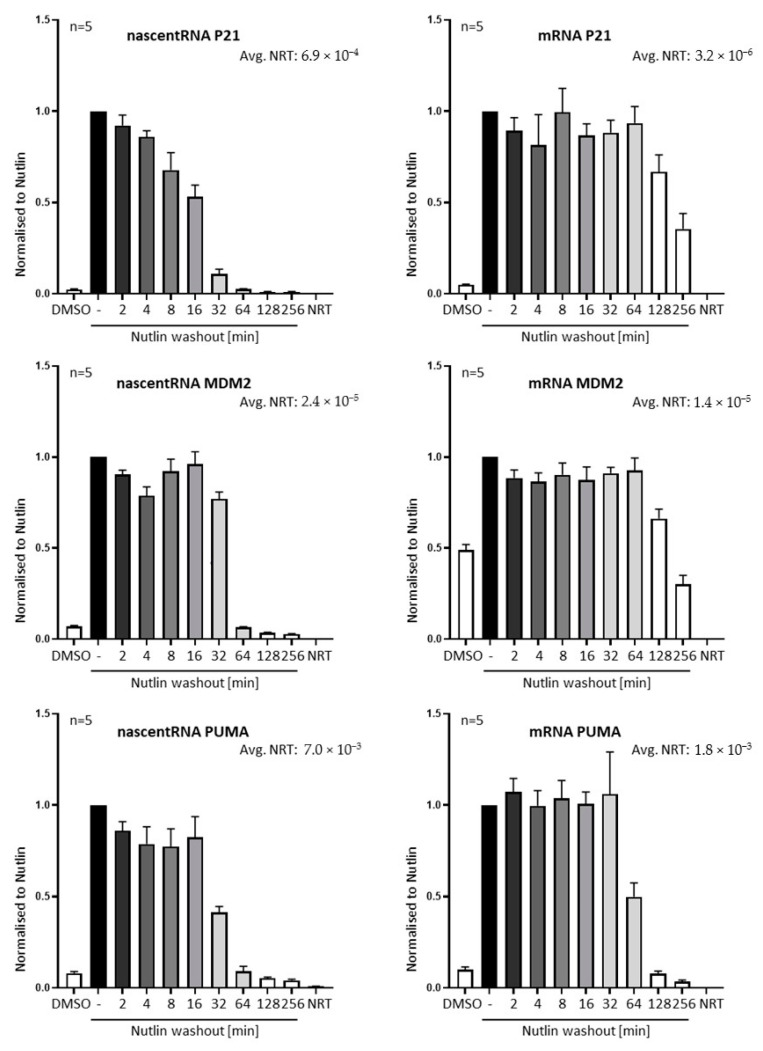
Rapid reduction in transcription from p53-responsive genes upon Nutlin removal. SJSA cells were treated with Nutlin, followed by wash-out for the indicated periods. RNA was isolated and analyzed by reverse transcription and qPCR. Quantification of non-processed pre-mRNA (nascent RNA) and processed mRNA of the target genes P21, MDM2 and PUMA reflected the increase in transcriptional activity upon Nutlin treatment and its reduction after Nutlin washout. From five biological replicates, the RNA levels were normalized to the amount of RNA from the housekeeping gene 36B4, followed by normalization to the Nutlin-treated sample before washout, and displayed as mean + SEM. NRT controls reflect the negligeable amount of DNA contamination of all RNA samples. The reduction of RNA synthesis (nascent RNA) was seen within less than one hour after washout. “-“ indicates that Nutlin was not washed out before harvest.

The displacement of p53 from promoters was followed by a drop in transcription of p53-responsive genes, as judged by the quantification of incompletely spliced pre-mRNA (nascent RNA) by RT-PCR (Figure 2). For appropriate controls, the samples were treated with DNase before reverse transcription (RT), and a no-RT control was included in the experiment. Interestingly, the velocity of the depletion of pre-mRNA seems to differ between the target genes, whereas the removal of pre-bound p53 from promoter DNA was quite uniform (Figure 1F).

This can hint to a variance in the lifespan of different pre-mRNAs or to additional levels of transcriptional regulation, following the displacement of p53 from promoters, at least in SJSA cells.

### 3.3. P53 Removal from Promoters Strictly Depends on MDM2

**Figure 3:**
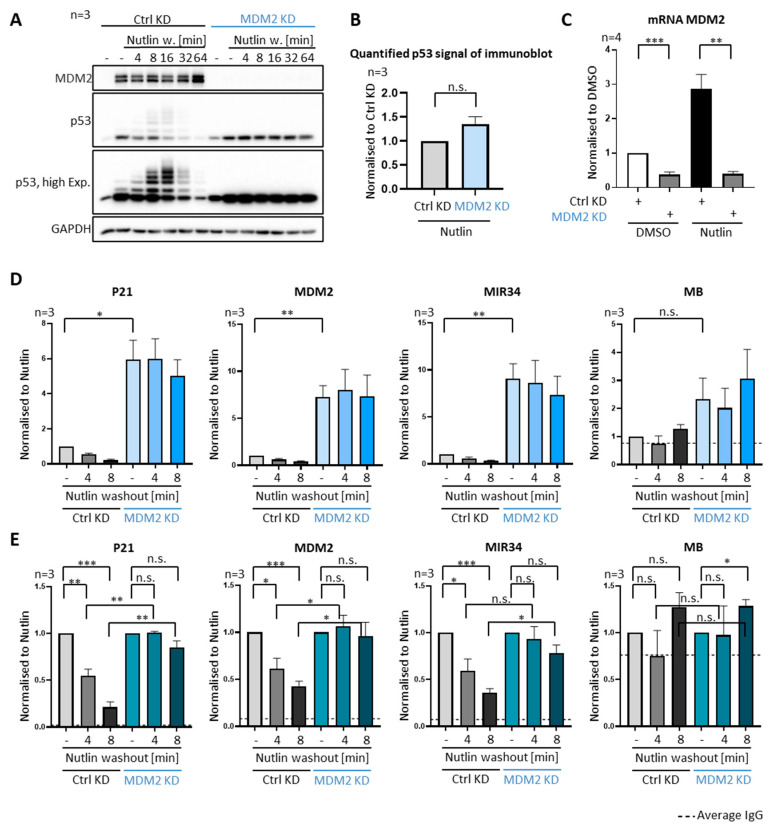
Requirement of MDM2 for removing p53 from promoters. (**A**–**E**) The Nutlin washout experiment was preceded by the depletion of MDM2 using siRNA transfection for 48 h, or by transfection of a scrambled negative control siRNA. (**A**) Knockdown (KD) of MDM2 largely abolished p53 ubiquitination and decelerated its degradation. (**B**) Quantification of the signal corresponding to p53 upon Nutlin treatment and either control KD or MDM2 KD in three biological replicates of immunoblot analyses, as shown in (**A**), lanes 2 and 8. Upon Nutlin treatment, p53 levels are hardly rising in cells transfected with siRNA to MDM2. (**C**) mRNA of MDM2 was measured by qPCR to confirm the depletion of MDM2 by siRNA transfection. (**D**) ChIP with anti-p53 antibodies after MDM2 depletion and Nutlin washout. P53 occupancy at its target genes P21, MDM2, MIR34 and MB (negative control) revealed increased p53 occupancy upon MDM2 depletion, and diminished removal of p53 upon Nutlin washout. Three biological replicates were normalized to chromatin input and then to the control transfected Nutlin sample (sample 1; without washout) and shown as mean + SEM. MDM2 depletion strongly increased the association of p53 with promoter DNA. (**E**) The same data as in (**D**) are now displayed with both Nutlin samples (samples 1 and 4; each prior to washout), regardless of the siRNA, set to 1 and used as normalization references for each siRNA species. This normalization allows a better comparison of both Nutlin washouts regarding their impact on the relative loss of p53 on promoters. Knockdown of MDM2 increased the stable association of p53 with its cognate promoter elements. *** *p* < 0.001; ** *p* < 0.01; * *p* < 0.05; n.s., non-significant. “-“, no Nutlin washout.

To ensure that MDM2 is indeed required for the ubiquitination of p53 and its removal from promoters in our assay system, we repeated the Nutlin washout experiment after transfecting the cells with siRNA to knock down MDM2, side by side to cells transfected with non-targeting control siRNAs. As expected, p53 was not detectably ubiquitinated upon MDM2 knockdown (Figure 3A,C) and remained stably associated with promoters, regardless of Nutlin washout, corroborating the role of MDM2 in removing p53 (Figure 3D,E).

We also noted that the levels of DNA-associated p53 were considerably higher upon MDM2 knockdown (Figure 3D, compare lane 1 to lane 4), although the overall amounts of p53 in Nutlin-treated SJSA cells remained grossly similar regardless of the siRNA treatment (Figure 3A, compare lane 2 to lane 8, quantification in Figure 3B). We speculate that the high amounts of MDM2 that accumulated upon Nutlin treatment can impair the association of p53 with DNA, even if the primary interaction of the amino–terminal domains is obliterated. Perhaps, the additional interactions, e.g., between MDM2 and the carboxyterminal regions of p53 [21], can still confer some antagonism of the p53–DNA association.

### 3.4. P53 Removal from Promoters Does Not Require Proteasomal Activity

**Figure 4:**
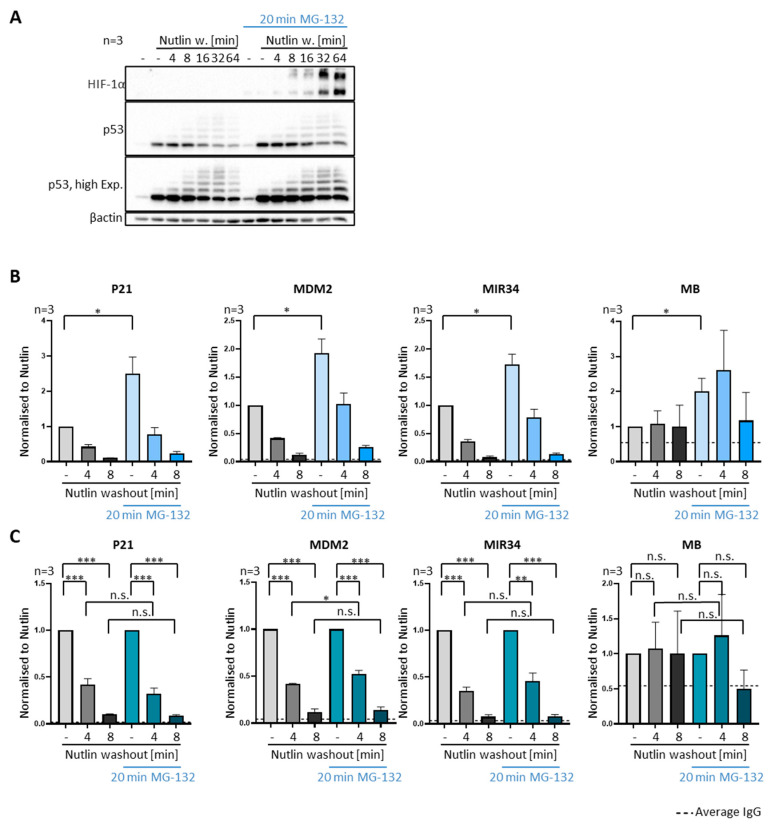
Removal of p53 from promoters independent of proteasome activity. (**A**–**C**) To inhibit the proteasome for a brief period, SJSA cells were treated with the pharmacological proteasome inhibitor 20 µM MG-132, or DMSO alone, for 20 min prior to Nutlin washout. Proteasome inhibition was continued after Nutlin was removed. (**A**) The delayed degradation of p53 as well as the accumulation of ubiquitinated p53 upon proteasome inhibition is visible in representative immunoblots. HIF-1α, a protein with particularly high turnover [22], was detected to control proteasome inhibition. (**B**) A brief pulse of MG-132 increases the amount of DNA-bound p53 but does not compromise p53-removal from promoters upon Nutlin washout, as revealed by ChIP analysis. Three biological replicates, mean + SEM. (**C**) The same data as in (**B**) but normalized as described for Figure 3E, visualizing comparable removal of p53 from its cognate promoters regardless of short proteasome inhibition. *** *p* < 0.001; ** *p* < 0.01; * *p* < 0.05; n.s., non-significant. “-“, no Nutlin washout.

MDM2 is a ubiquitin ligase that triggers the proteasomal degradation of p53. We therefore asked whether the proteasome is involved in the removal of p53 from promoters by MDM2. To test this, we added the proteasome inhibitor MG-132 to SJSA and MCF7 cells 20 min prior to Nutlin washout and then throughout the experiment until cell harvest. Although MG-132 led to the increased p53 levels (Figure 4A and Figure S2A) and occupancy at its target genes (Figure 4B and Figure S2B), it did not compromise the removal of p53 from promoters (Figure 4C and Figure S2C). To ensure proteasome inhibition by MG-132, we detected the accumulation of HIF-1α (Figure 4A), a protein that undergoes rapid degradation when proteasomes are active [22]. By calculating the ratio of the p53-associated promoter DNA before and after Nutlin washout, separately for the presence and absence of MG-132, it became obvious that the velocity of the removal was not grossly changed by proteasome inhibition (Figure 4C and Figure S2C). Thus, proteasome-mediated proteolytic activity is not required for the ability of MDM2 to dissociate p53 from its cognate promoter elements.

### 3.5. DNA Damage Does Not Compromise Dissociation of Prebound P53 from Promoter Sites

**Figure 5:**
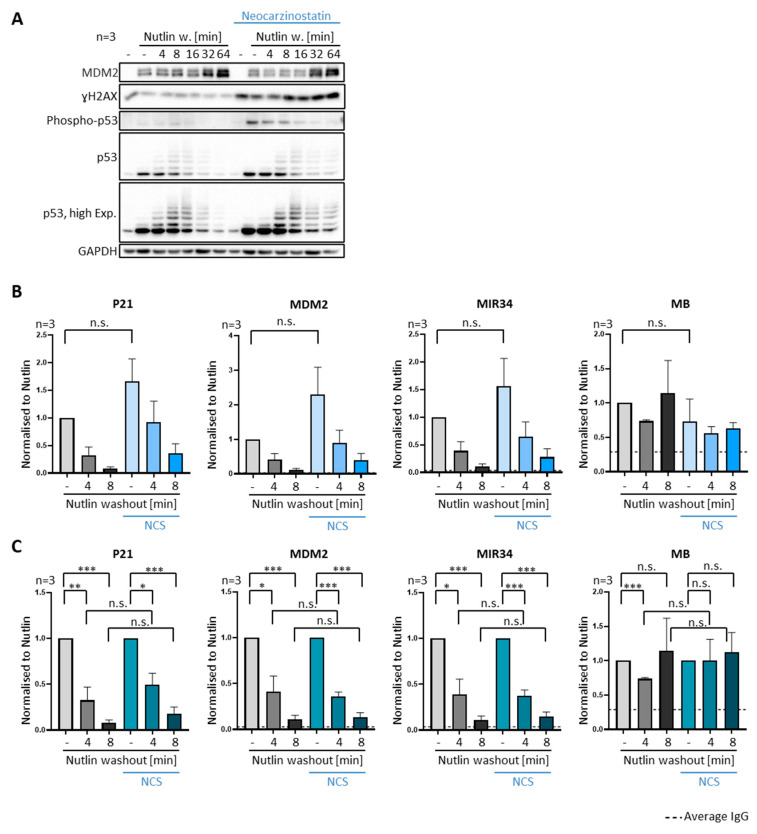
Removal of p53 regardless of DNA damage signaling. (**A**–**C**) SJSA cells were treated with 250 ng/mL of neocarzinostatin (NCS), a drug that induces double-strand breaks (DSBs) in cellular DNA. Treatment was started 2 h prior to Nutlin washout and was continued after Nutlin was removed. (**A**) Immunoblot analysis revealed the expected increase in the DNA damage marker γH2AX and enhanced phosphorylated p53 at Serin 20. (**B**) The association of p53 with chromatin in SJSA cells was investigated by ChIP. P53 dissociated from its cognate promoters upon Nutlin washout regardless of NCS treatment. (**C**) Data as in (**B**) but normalized as described in Figure 3E. *** *p* < 0.001; ** *p* < 0.01; * *p* < 0.05; n.s., non-significant. “-“, no Nutlin washout.

We also asked if DNA damage signaling can interfere with the removal of p53 from promoters. To this end, we treated SJSA cells with neocarzinostatin (NCS), a radiomimetic inducer of double strand DNA breaks [23]. As expected, neocarzinostatin led to the quick accumulation of phosphorylated histone H2AX, as part of the DNA damage response. It also reduced p53 degradation and thus increased the levels of both unmodified and ubiquitinated p53, as well as phosphorylated p53 (Figure 5A). However, neocarzinostatin did not compromise the velocity by that p53 was removed from promoters (Figure 5B,C). Despite the previously described impact of the DNA damage response on p53 activation [24,25] and MDM2 inhibition [19,26,27], this does not seem to reduce the ability of MDM2 to remove p53 from promoters, at least in SJSA cells.

### 3.6. Ubiquitin Is Required for the Removal of P53 from Promoters

**Figure 6:**
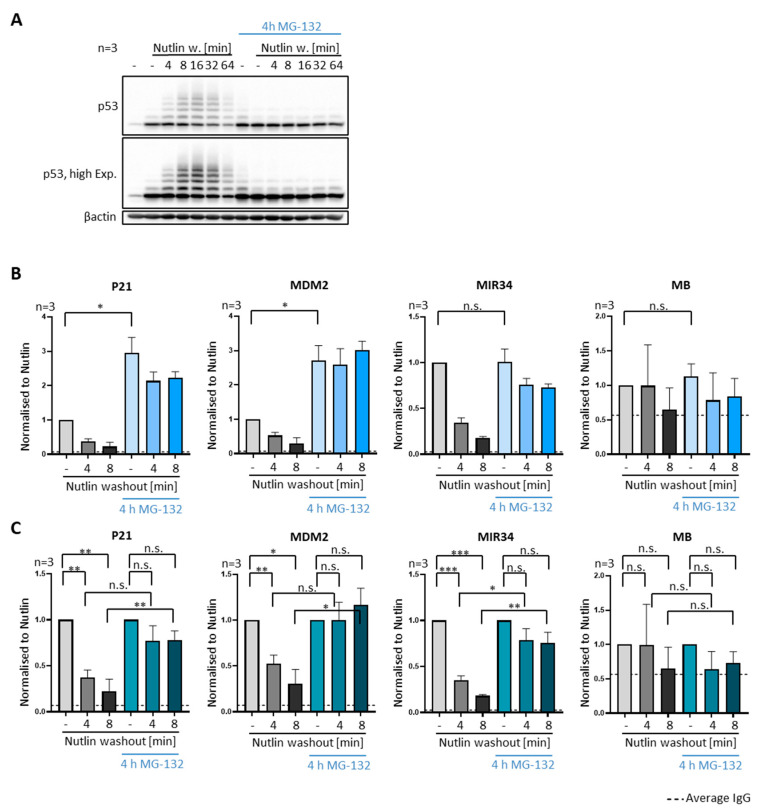
Requirement of ubiquitin for removing p53 from promoters. (**A**–**C**) To deplete ubiquitin, SJSA cells were treated for 4 h with the proteasome inhibitor MG-132 (20 µM). This treatment interrupts the recycling of ubiquitin from ubiquitin-conjugated proteins and thus precludes de novo ubiquitination. (**A**) Prolonged proteasome inhibition largely abolishes the ubiquitination of p53, as observed by immunoblot analysis. (**B**,**C**) P53 occupancy at target gene promoters, as measured by ChIP analysis, is significantly higher after prolonged MG-132 treatment, and there is no significant reduction upon Nutlin washout. *** *p* < 0.001; ** *p* < 0.01; * *p* < 0.05; n.s., non-significant. “-”, no Nutlin washout.

We then tested in SJSA and MCF7 cells whether ubiquitination is involved in the removal of p53 from promoters, as suggested by the similar timing patterns of ubiquitination and promoter dissociation (Figure 1). We first used a prolonged incubation with MG-132, i.e., for 4 h prior to Nutlin washout, to deplete the cells of ubiquitin. Such long periods of proteasome inhibition are known to eliminate ubiquitin monomers from the cell, presumably by the accumulation of ubiquitinated proteins that are not degraded, thus precluding the recycling of free ubiquitin [28]. Indeed, we observed by immunoblot analysis that the treatment with MG-132 for 4 h led to the accumulation of some ubiquitinated p53, compatible with proteins that are ubiquitinated but not degraded upon proteasome inhibition (Figure 6A and Figure S3A, compare lane 1 and 8, respectively). Even more remarkably, however, the same immunoblots revealed that, by treating the cells with Nutlin simultaneously to prolonged proteasome inhibition, MDM2-dependent ubiquitination of p53 was largely abolished.

In agreement, we did not observe p53 ubiquitination after Nutlin washout and prolonged proteasome inhibition even though MDM2 was reactivated, conceivably because ubiquitin monomers are eliminated from the cells under those conditions. Strikingly, this treatment also severely compromised or even abolished the removal of p53 from the target gene promoters upon Nutlin washout (Figure 6B,C and Figure S3B,C). This is in stark contrast to the effect of a short MG-132 treatment that is sufficient for proteasome inhibition but not for ubiquitin depletion, which had no impact on p53-removal from its target gene promoters (Figure 4B,C and Figure S2B,C).

### 3.7. Inhibiting E1 Ubiquitin Ligases Delays the Removal of P53 from Promoters

**Figure 7:**
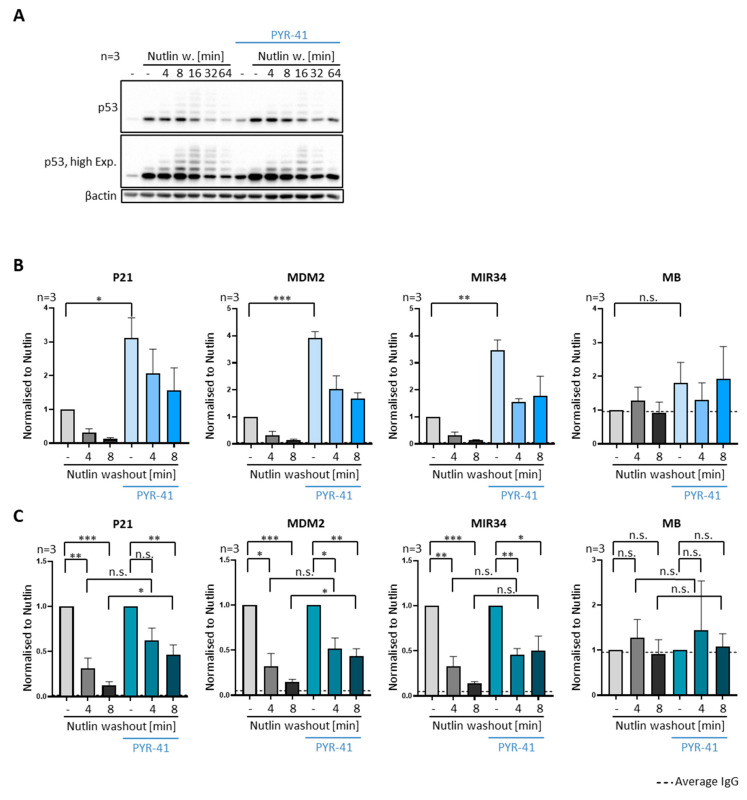
Attenuated p53-removal upon E1 ligase inhibition. (**A**–**C**) Ubiquitination was inhibited by adding 20 µM of the E1 ubiquitin activating enzyme inhibitor PYR-41 for 4 h prior to Nutlin washout, with continued treatment after Nutlin removal. (**A**) Immunoblot analysis revealed that p53 ubiquitination and degradation after Nutlin washout is reduced by PYR-41, albeit to a lesser extent than upon prolonged proteasome inhibition (Figure 6A). (**B**) P53 occupancy on promoter DNA, detected by ChIP analysis, was significantly increased upon treatment with PYR-41. The velocity of p53 removal, however, was reduced by PYR-41, as further visualized in (**C**). *** *p* < 0.001; ** *p* < 0.01; * *p* < 0.05; n.s., non-significant. “-“, no Nutlin washout.

We also assessed the removal of p53 from promoters in the presence of an E1 ligase inhibitor, PYR-41, in SJSA cells [29]. PYR-41 treatment also obliterated both the ubiquitination of p53 (Figure 7A) and its removal from promoter DNA upon Nutlin washout (Figure 7B,C), albeit both to a lesser extent than through ubiquitin depletion by prolonged MG-132 treatment.

In conclusion, the availability of transferable ubiquitin is a prerequisite not only for p53 ubiquitination, but also for the dissociation of p53 from chromatin by MDM2.

### 3.8. MDM4 Is Not Required for Removing P53 from Promoters, despite Its Requirement for Polyubiquitination

MDM4 not only interacts with MDM2 and p53, but it is also an essential p53 antagonist in vivo [3]. Hence, we sought to determine if MDM4 is also involved in the removal of p53 from its target gene promoters. Upon the knockdown of MDM4, followed by Nutlin treatment and washout, we found a reduced overall ubiquitination of p53 in the SJSA cells (Figure 6A). It was reported that MDM2 alone is needed to monoubiquitinate p53, while MDM4 and MDM2 are needed for polyubiquitinating p53 [7,30], although we only observed a relatively mild reduction in the levels of p53 with multiple ubiquitin moieties upon MDM4 depletion. Strikingly, however, the depletion of MDM4 did not affect the rapid removal of p53 from promoter DNA (Figure 6C,D), in stark contrast with the impact of MDM2 depletion (Figure 3C,D). Thus, MDM4 is not required for dissociating p53 from promoters, while it is contributing to its efficient polyubiquitination. This at least suggests that p53 only needs to be ubiquitinated to a moderate extent by MDM2 in order to separate p53 from its cognate DNA elements.

## 4. Discussion

**Figure 8:**
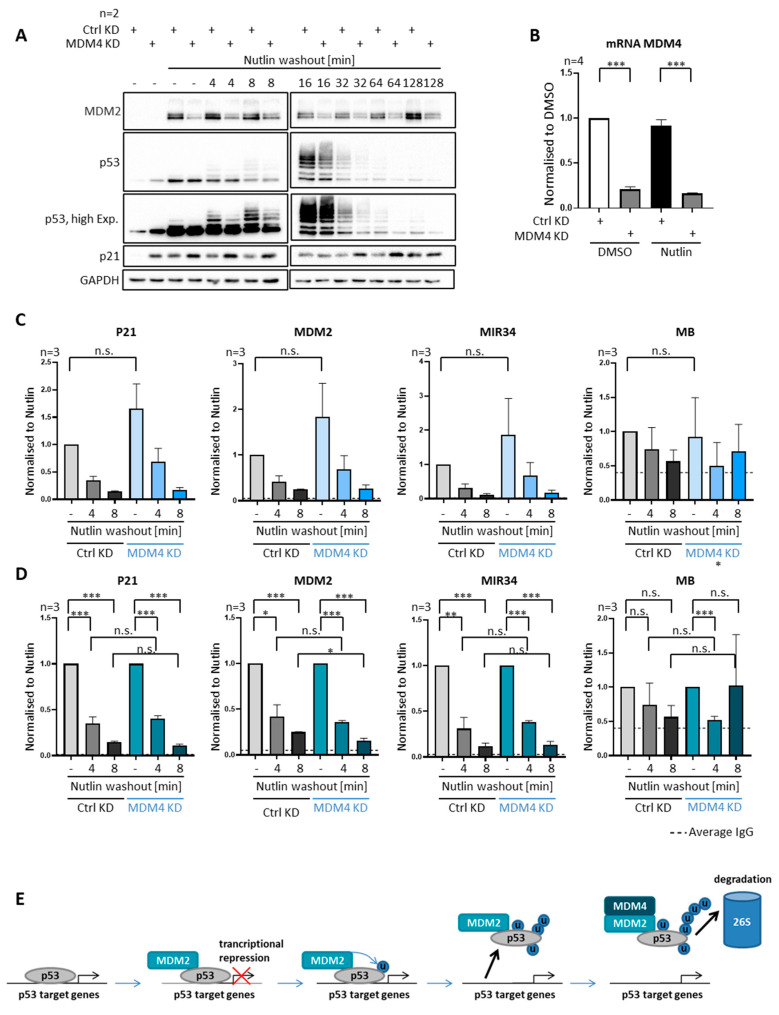
Efficient removal of p53 from promoters upon depletion of MDM4. (**A**–**D**) SJSA cells were transfected with siRNA against MDM4 48 h prior to Nutlin washout, or control transfected. (**A**) Depletion of MDM4 leads to the reduced ubiquitination of p53 and increased induction of p21 as visible by the analysis of a representative immunoblot. (**B**) Measurement of MDM4 mRNA shows an efficient MDM4 depletion by siRNA treatment. (**C**) ChIP analysis upon Nutlin washout, with or without MDM4 depletion. (**D**) The dissociation of p53 from promoters upon Nutlin washout was still significant and did not grossly differ regardless of MDM4 depletion. (**E**) Graphical summary of the proposed mechanism of MDM2-dependent p53 removal from cognate promoter sites. MDM2 associates with p53 and inhibits its transcriptional activity. MDM2-dependent ubiquitination of p53 is a necessary prerequisite for p53 removal from its target genes. Subsequently, the MDM2–MDM4 complex polyubiquitinates p53, followed by its proteasomal degradation. *** *p* < 0.001; ** *p* < 0.01; * *p* < 0.05; n.s., non-significant. “-“, no Nutlin washout.

Our results strongly suggest that MDM2-mediated ubiquitination not only triggers p53-degradation, but also the removal of p53 from its association with promoter-DNA within minutes. While the velocity of the removal seems to differ between cell lines, the dependency on MDM2-mediated ubiquitination, but not proteasome-mediated degradation, was found in cells with and without MDM2 amplification. While MDM2 is strictly required for this removal, MDM4 is not (Figure 3 and Figure 8).

How can the transfer of ubiquitin onto p53 interfere with DNA binding? Most ubiquitination sites within p53 reside within the carboxyterminal domain [1,31], which mediates the tetramerization of p53 but is not directly involved in DNA binding. On the one hand, avidity effects strongly contribute to the interaction of the central domains of a p53 tetramer with the DNA, and this is reflected by the fact that most p53-responsive promoter elements contain four half-sites that each interact with one central domain of p53 [32]. Thus, disrupting oligomerization can abolish such cooperative binding and, hence, loosen the association between p53 and DNA. On the other hand, previous reports argue that ubiquitination does not compromise oligomerization of p53, whereas it does diminish DNA binding in vitro [12]. Thus, ubiquitination can trigger changes in the conformation of the tetramer without disrupting it, e.g., by reducing the flexibility of the linkers between oligomerization domains and DNA binding domains.

We found that neither the depletion of MDM4 nor the treatment with the dsDNA break-inducing agent neocarzinostatin compromised the removal of p53 from promoter DNA by MDM2. In parallel, the absence of MDM4 mostly affects polyubiquitination but not monoubiquitination of p53 [7,30]. Interestingly, the phosphorylation of MDM2 by the principal kinase induced by dsDNA breaks, ATM, was also reported to reduce the polyubiquitination of p53 much more than monoubiquitination [19]. These results from the literature, taken together with our findings, at least suggest that the addition of one or a few ubiquitin moieties to p53 is enough to dissociate it from promoters, with little if any impact of subsequent polyubiquitination. However, this model awaits confirmation by more detailed analyses.

What could be the biological significance of removing p53 from promoters prior to its degradation? We propose that three consequences led to the evolution of this mechanism.

Firstly, the rapid detachment of p53 from the DNA almost instantaneously reduces its ability to induce transcription of its target genes—we have observed this by quantifying non-processed pre-mRNA corresponding to p53 target genes (Figure 2). This conceivably avoids prolonged cell cycle arrest or excessive cell death, e.g., in situations when damaged DNA is repaired and the quick proliferation of the cell is required. 

Secondly, the induced dissociation of p53 from promoters can represent an additional level of control in situations where the proteasomal activity is reduced. Proteasome activity is subject to regulation on its own, e.g., through the miRNA-regulated chaperone POMP [33], but the control on p53 is required for cell survival even when proteasome activity is low. This can be enabled by limiting the association of p53 with DNA, rather than relying on its destabilization. 

Thirdly, when a cell contains sufficient proteasomal activity, the removal of p53 from chromatin can accelerate and facilitate its subsequent degradation. A large proportion of proteasome activity is found in the cytosol, and even the nuclear proteasomes are not typically associated with chromatin, especially in dividing cells [9,10]. This can even make it necessary to export p53 and MDM2 from the nucleus to the cytosol for efficient degradation [12,34,35,36]. In any case, p53 would obviously need to dissociate from chromatin for reaching either the proteasomes within the nucleus or the nuclear export machinery. Thus, the removal of p53 from promoters by MDM2 not only allows for quick negative regulation of its activity but can constitute a prerequisite for its efficient proteasomal degradation.

On the other hand, removing p53–MDM2 complexes from chromatin conceivably precludes the formation of repressive complexes, as has been suggested in early work [14]. Turning an activator to a repressor is a common theme, otherwise, in transcriptional regulation, e.g., when the transcriptional activator E2F1 associates with members of the retinoblastoma protein family [37]. The resulting complexes typically remain on the chromatin and now act as repressors of transcription, e.g., by recruiting HDACs. Our results suggest that p53 cannot be employed to form repressive complexes but merely loses its ability to activate promoters when bound to MDM2.

The question remains whether this activity of MDM2, i.e., its ability to dissociate p53 from DNA, can be regulated under physiological circumstances. For instance, the acetylation of p53 at lysine residues will preclude the ubiquitination of these residues and can thus stabilize the interaction of p53 with promoter DNA—and indeed, histone acetyl transferases do act as stabilizers and activators of p53 [38]. The same can apply to the phosphorylation of MDM2, which sometimes activates its ubiquitin ligase activity towards p53, an example being the AKT-driven phosphorylation of the residues Ser166 and Ser186 within MDM2 [39,40]. It is tempting to speculate that many regulatory pathways acting on p53 and MDM2 for p53 ubiquitination may not only affect the degradation of p53, but also directly govern the association of p53 with DNA.

## Figures and Tables

**Table 1:** Primer sequences for targeted ChIP.

Primer Target	Forward (5′-3′)	Reverse (5′-3′)
P21	CTTTCTGGCCGTCAGGAACA	CTTCTATGCCAGAGCTCAACATGT
MDM2	TTCAGTGGGCAGGTTGACTC	CCAGCTGGAGACAAGTCAGG
MIR34	ATTCTTCCCCTTACGGAGGC	GAAGGAGGCGGGAACTAGAC
PUMA	CCCTGCTCTGGTTTGGTGAG	AGTCACTCTGGTGAGGCGAT
PIG3	CCCTGGGTACCTGCATTAAG	TAGCCGTGCACTTTGACAAG
MB	CTCATGATGCCCCTTCTTCT	GAAGGCGTCTGAGGACTTAAA

**Table 2:** Primer sequences for RNA quantification.

Primer Target	Forward (5′-3′)	Reverse (5′-3′)
nascent RNA P21	AGACCAGCATGACAGGTGCG	GCCTGGCATAATGAACATTCCCA
mRNA P21	TAGGCGGTTGAATGAGAGG	AAGTGGGGAGGAGGAAGTAG
nascent RNA MDM2	CATTGGTTTGTGGACTTGAGGAT	TAAAGGCAGTCACTTCTGGGA
mRNA MDM2	CCGGATTAGTGCGTACGAG	GTCTCTTGTTCCGAAGCTGGA
nascent RNA PUMA	ATTTCCGGGTGCGCTCT	CCTCAACACTCCCTAGCAACT
mRNA PUMA	GACGACCTCAACGCACAGTA	TAATTGGGCTCCATCTCGGG
mRNA MDM4	CTCAGACTCTCGCTCTCGCA	CTCAAATCCAAGGTCCAGCC
mRNA 36B4	GATTGGCTACCCAACTGTTG	CAGGGGCAGCAGCCACAAA

## Supplementary Materials

The following is available online at https://www.mdpi.com/article/10.3390/biom12010022/s1, Figure S1: rapid removal of p53 upon Nutlin washout occurs in two additional cell lines. Figure S2: rapid removal of p53 from promoters does not require proteasome activity in MCF7 cells. Figure S3: depletion of ubiquitin by prolonged proteasome inhibition halts p53 removal in MCF7 cells. Figure S4: rapid removal of p53 upon Nutlin washout is observed with different anti-p53 antibodies used for ChIP. Figure S5: p53 antibodies used for ChIP and immunoblot analyses precipitate ubiquitinated p53.
