# Supplementary material for: MDM2-Driven Ubiquitination Rapidly Removes p53 from Its Cognate Promoters
Source: Biomolecules. 2021 Dec 24;12(1):22. doi: 10.3390/biom12010022 (PMC8773640; doi:10.3390/biom12010022)
Supplement: Supplementary file 1 [file biomolecules-12-00022-s001.zip › biomolecules-1437984-supplementary.pptx]

## Slide 1
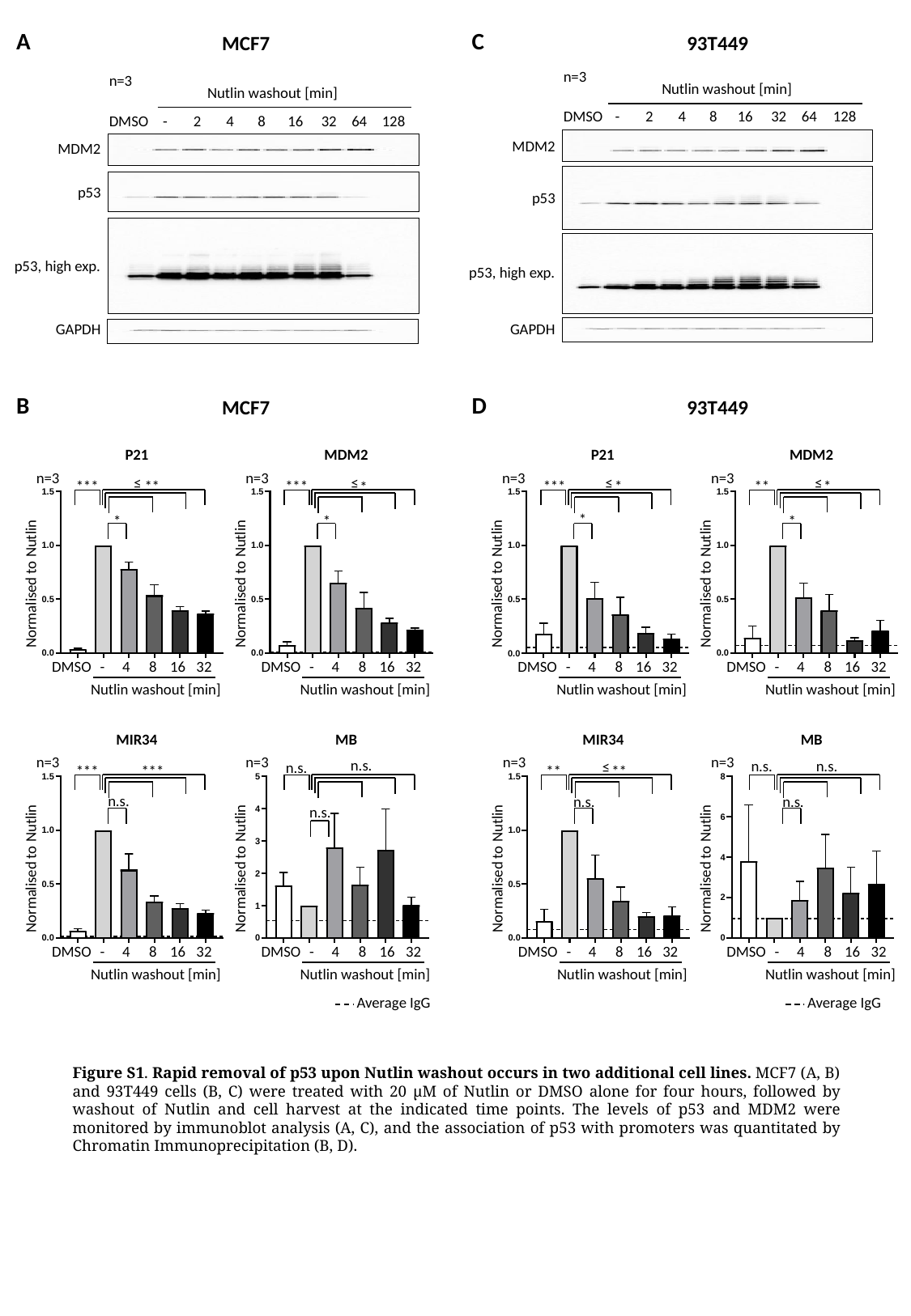

A
C
MCF7
93T449
n=3
Nutlin washout [min]
DMSO
-
2
4
8
16
32
64
128
MDM2
p53
p53, high exp.
 GAPDH
n=3
Nutlin washout [min]
DMSO
-
2
4
8
16
32
64
128
MDM2
p53
p53, high exp.
 GAPDH
B
D
MCF7
93T449
P21
n=3
***
**
*
 Normalised to Nutlin
DMSO
-
4
8
16
32
Nutlin washout [min]
MDM2
n=3
***
*
*
 Normalised to Nutlin
DMSO
-
4
8
16
32
Nutlin washout [min]
MDM2
n=3
**
*
*
 Normalised to Nutlin
DMSO
-
4
8
16
32
Nutlin washout [min]
P21
n=3
*
***
*
 Normalised to Nutlin
DMSO
-
4
8
16
32
Nutlin washout [min]
≤
≤
≤
≤
MIR34
n=3
***
***
n.s.
 Normalised to Nutlin
DMSO
-
4
8
16
32
Nutlin washout [min]
MB
n=3
n.s.
 Normalised to Nutlin
DMSO
-
4
8
16
32
Nutlin washout [min]
MIR34
n=3
**
**
n.s.
 Normalised to Nutlin
DMSO
-
4
8
16
32
Nutlin washout [min]
MB
n=3
n.s.
n.s.
n.s.
 Normalised to Nutlin
DMSO
-
4
8
16
32
Nutlin washout [min]
n.s.
≤
n.s.
Average IgG
Average IgG
Figure S1. Rapid removal of p53 upon Nutlin washout occurs in two additional cell lines. MCF7 (A, B) and 93T449 cells (B, C) were treated with 20 µM of Nutlin or DMSO alone for four hours, followed by washout of Nutlin and cell harvest at the indicated time points. The levels of p53 and MDM2 were monitored by immunoblot analysis (A, C), and the association of p53 with promoters was quantitated by Chromatin Immunoprecipitation (B, D).

## Slide 2
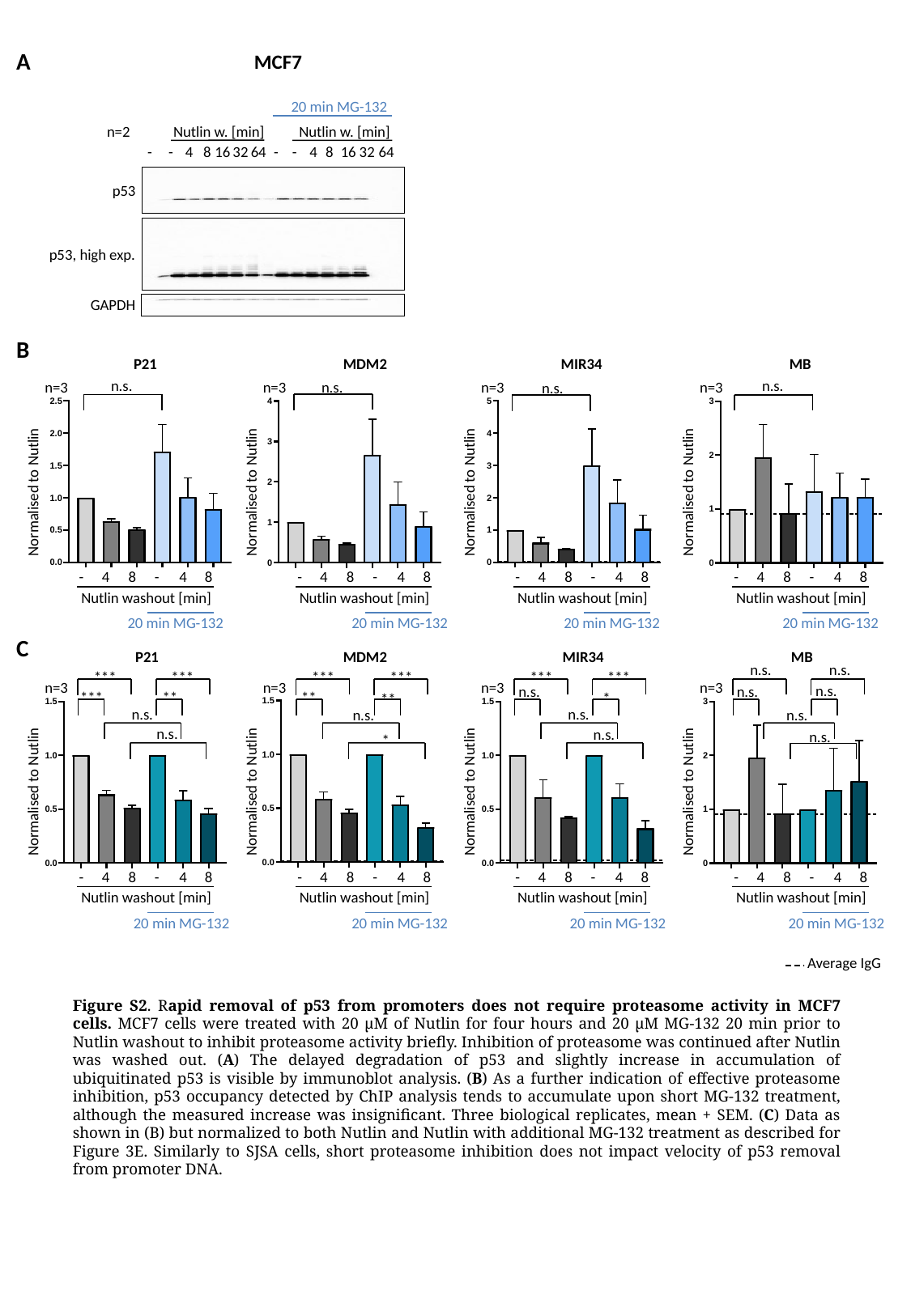

A
MCF7
20 min MG-132
Nutlin w. [min]
Nutlin w. [min]
n=2
-
-
4
8
16
32
64
-
-
4
8
16
32
64
p53
p53, high exp.
 GAPDH
B
P21
n.s.
n=3
 Normalised to Nutlin
-
4
8
-
4
8
Nutlin washout [min]
20 min MG-132
MDM2
n=3
n.s.
 Normalised to Nutlin
-
4
8
-
4
8
Nutlin washout [min]
20 min MG-132
MIR34
n=3
n.s.
 Normalised to Nutlin
-
4
8
-
4
8
Nutlin washout [min]
20 min MG-132
MB
n.s.
n=3
 Normalised to Nutlin
-
4
8
-
4
8
Nutlin washout [min]
20 min MG-132
C
P21
MDM2
***
***
n=3
**
**
n.s.
*
-
4
8
-
4
8
Nutlin washout [min]
20 min MG-132
 Normalised to Nutlin
MIR34
***
***
n=3
n.s.
*
n.s.
n.s.
 Normalised to Nutlin
-
4
8
-
4
8
Nutlin washout [min]
20 min MG-132
MB
n.s.
n.s.
***
***
n=3
n=3
n.s.
n.s.
***
**
n.s.
n.s.
n.s.
n.s.
 Normalised to Nutlin
 Normalised to Nutlin
-
4
8
-
4
8
-
4
8
-
4
8
Nutlin washout [min]
Nutlin washout [min]
20 min MG-132
20 min MG-132
Average IgG
Figure S2. Rapid removal of p53 from promoters does not require proteasome activity in MCF7 cells. MCF7 cells were treated with 20 µM of Nutlin for four hours and 20 µM MG-132 20 min prior to Nutlin washout to inhibit proteasome activity briefly. Inhibition of proteasome was continued after Nutlin was washed out. (A) The delayed degradation of p53 and slightly increase in accumulation of ubiquitinated p53 is visible by immunoblot analysis. (B) As a further indication of effective proteasome inhibition, p53 occupancy detected by ChIP analysis tends to accumulate upon short MG-132 treatment, although the measured increase was insignificant. Three biological replicates, mean + SEM. (C) Data as shown in (B) but normalized to both Nutlin and Nutlin with additional MG-132 treatment as described for Figure 3E. Similarly to SJSA cells, short proteasome inhibition does not impact velocity of p53 removal from promoter DNA.

## Slide 3
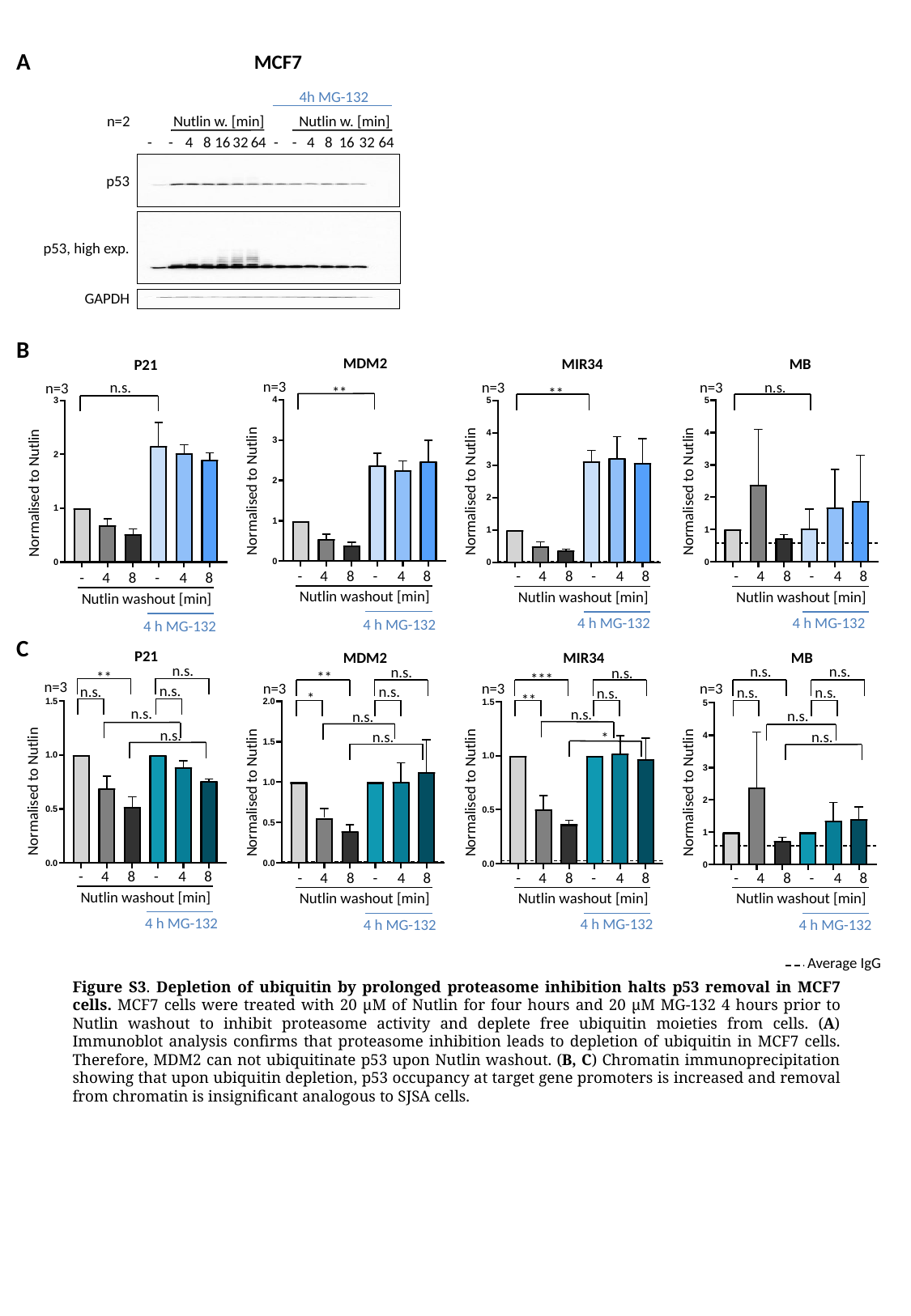

A
MCF7
4h MG-132
Nutlin w. [min]
Nutlin w. [min]
n=2
16
-
-
4
8
16
32
64
-
-
4
8
32
64
p53
p53, high exp.
 GAPDH
B
MDM2
n=3
**
 Normalised to Nutlin
-
4
8
-
4
8
Nutlin washout [min]
4 h MG-132
MIR34
n=3
**
 Normalised to Nutlin
-
4
8
-
4
8
Nutlin washout [min]
4 h MG-132
MB
n=3
n.s.
 Normalised to Nutlin
-
4
8
-
4
8
Nutlin washout [min]
4 h MG-132
P21
n.s.
n=3
 Normalised to Nutlin
-
4
8
-
4
8
Nutlin washout [min]
4 h MG-132
C
P21
n.s.
**
n=3
n.s.
n.s.
n.s.
n.s.
 Normalised to Nutlin
-
4
8
-
4
8
Nutlin washout [min]
4 h MG-132
MDM2
n.s.
**
n=3
n.s.
*
n.s.
 Normalised to Nutlin
-
4
8
-
4
8
Nutlin washout [min]
4 h MG-132
MIR34
n.s.
***
n=3
n.s.
**
 Normalised to Nutlin
-
4
8
-
4
8
Nutlin washout [min]
4 h MG-132
MB
n.s.
n.s.
n=3
n.s.
n.s.
n.s.
n.s.
 Normalised to Nutlin
-
4
8
-
4
8
Nutlin washout [min]
4 h MG-132
n.s.
n.s.
*
Average IgG
Figure S3. Depletion of ubiquitin by prolonged proteasome inhibition halts p53 removal in MCF7 cells. MCF7 cells were treated with 20 µM of Nutlin for four hours and 20 µM MG-132 4 hours prior to Nutlin washout to inhibit proteasome activity and deplete free ubiquitin moieties from cells. (A) Immunoblot analysis confirms that proteasome inhibition leads to depletion of ubiquitin in MCF7 cells. Therefore, MDM2 can not ubiquitinate p53 upon Nutlin washout. (B, C) Chromatin immunoprecipitation showing that upon ubiquitin depletion, p53 occupancy at target gene promoters is increased and removal from chromatin is insignificant analogous to SJSA cells.

## Slide 4
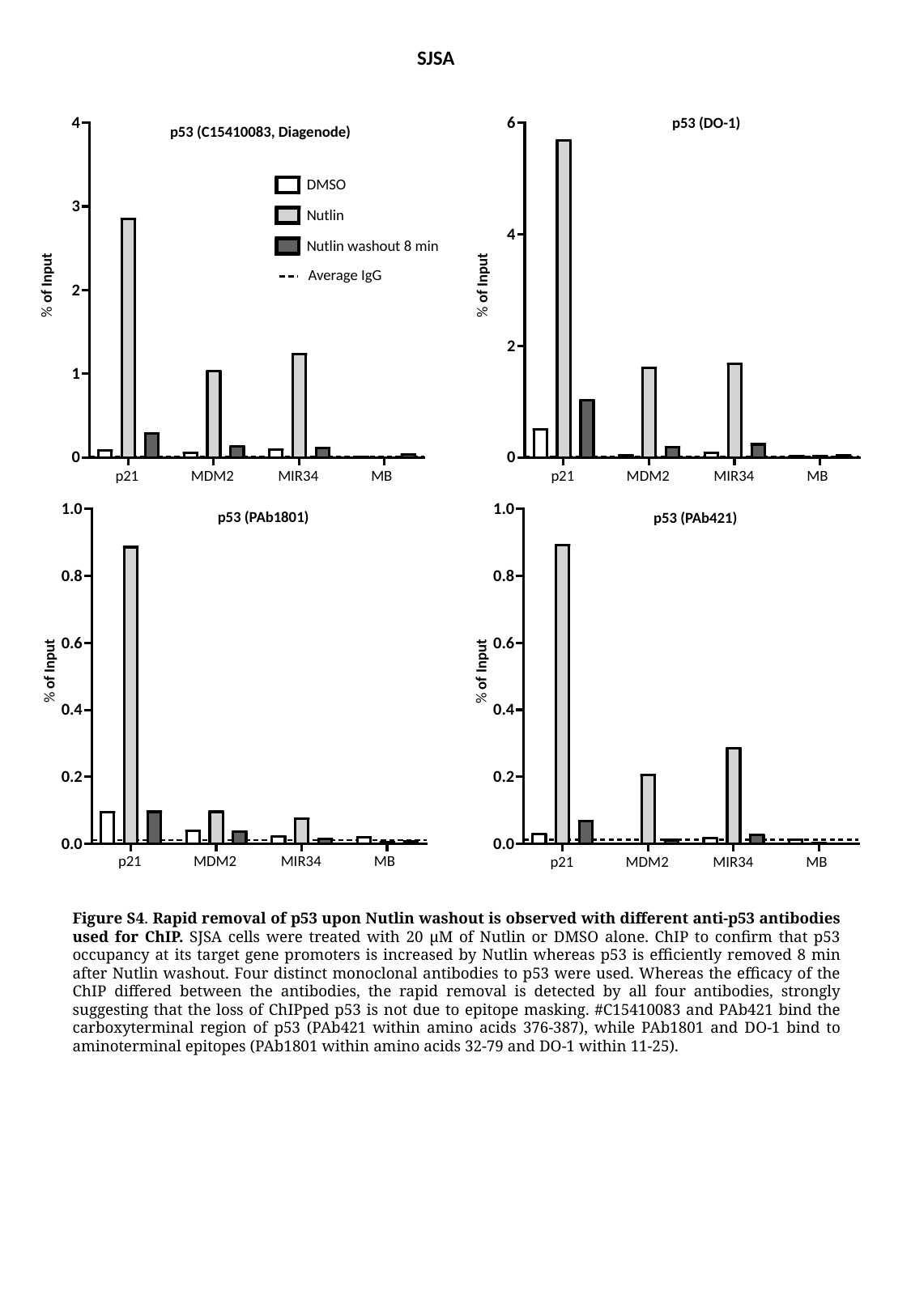

SJSA
p53 (DO-1)
p53 (C15410083, Diagenode)
DMSO
Nutlin
Nutlin washout 8 min
Average IgG
% of Input
% of Input
p21
MDM2
MIR34
MB
p21
MDM2
MIR34
MB
p53 (PAb1801)
p53 (PAb421)
% of Input
% of Input
p21
MDM2
MIR34
MB
p21
MDM2
MIR34
MB
Figure S4. Rapid removal of p53 upon Nutlin washout is observed with different anti-p53 antibodies used for ChIP. SJSA cells were treated with 20 µM of Nutlin or DMSO alone. ChIP to confirm that p53 occupancy at its target gene promoters is increased by Nutlin whereas p53 is efficiently removed 8 min after Nutlin washout. Four distinct monoclonal antibodies to p53 were used. Whereas the efficacy of the ChIP differed between the antibodies, the rapid removal is detected by all four antibodies, strongly suggesting that the loss of ChIPped p53 is not due to epitope masking. #C15410083 and PAb421 bind the carboxyterminal region of p53 (PAb421 within amino acids 376-387), while PAb1801 and DO-1 bind to aminoterminal epitopes (PAb1801 within amino acids 32-79 and DO-1 within 11-25).

## Slide 5
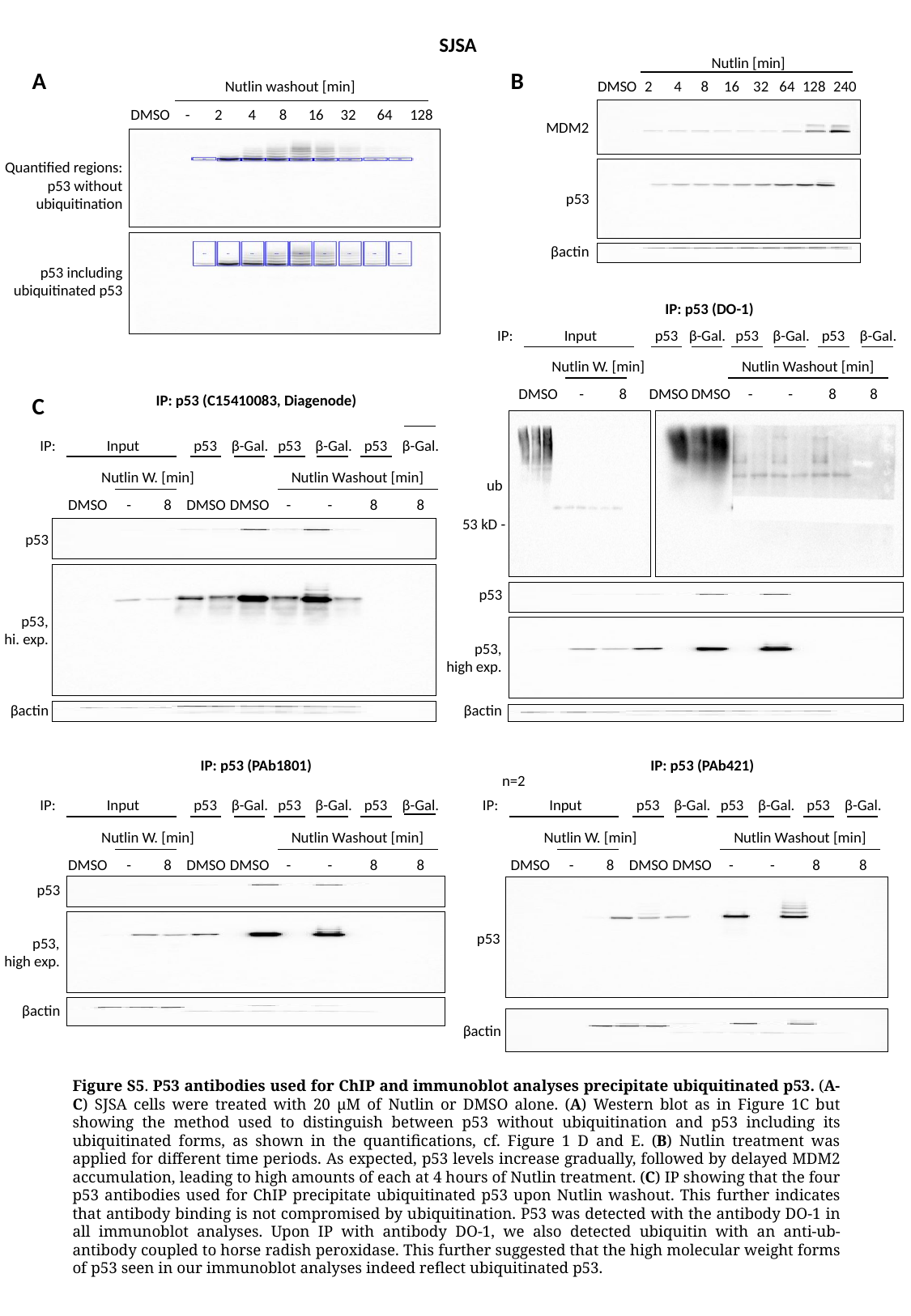

SJSA
Nutlin [min]
DMSO
2
4
8
16
32
64
128
240
MDM2
p53
 βactin
A
B
Nutlin washout [min]
DMSO
-
2
4
8
16
32
64
128
Quantified regions:
p53 without
ubiquitination
p53 including
ubiquitinated p53
IP: p53 (DO-1)
IP:
Input
p53
β-Gal.
p53
β-Gal.
p53
β-Gal.
Nutlin W. [min]
Nutlin Washout [min]
DMSO
DMSO
-
8
DMSO
-
-
8
8
IP: p53 (C15410083, Diagenode)
C
IP:
Input
p53
β-Gal.
p53
β-Gal.
p53
β-Gal.
Nutlin W. [min]
Nutlin Washout [min]
ub
DMSO
-
8
DMSO
DMSO
-
-
8
8
53 kD -
p53
p53
p53,
hi. exp.
p53,
high exp.
 βactin
 βactin
IP: p53 (PAb1801)
IP: p53 (PAb421)
n=2
IP:
Input
p53
β-Gal.
p53
β-Gal.
p53
β-Gal.
Nutlin W. [min]
Nutlin Washout [min]
DMSO
-
8
DMSO
DMSO
-
-
8
8
p53
p53,
high exp.
 βactin
IP:
Input
p53
β-Gal.
p53
β-Gal.
p53
β-Gal.
Nutlin W. [min]
Nutlin Washout [min]
DMSO
-
8
DMSO
DMSO
-
-
8
8
p53
 βactin
Figure S5. P53 antibodies used for ChIP and immunoblot analyses precipitate ubiquitinated p53. (A-C) SJSA cells were treated with 20 µM of Nutlin or DMSO alone. (A) Western blot as in Figure 1C but showing the method used to distinguish between p53 without ubiquitination and p53 including its ubiquitinated forms, as shown in the quantifications, cf. Figure 1 D and E. (B) Nutlin treatment was applied for different time periods. As expected, p53 levels increase gradually, followed by delayed MDM2 accumulation, leading to high amounts of each at 4 hours of Nutlin treatment. (C) IP showing that the four p53 antibodies used for ChIP precipitate ubiquitinated p53 upon Nutlin washout. This further indicates that antibody binding is not compromised by ubiquitination. P53 was detected with the antibody DO-1 in all immunoblot analyses. Upon IP with antibody DO-1, we also detected ubiquitin with an anti-ub-antibody coupled to horse radish peroxidase. This further suggested that the high molecular weight forms of p53 seen in our immunoblot analyses indeed reflect ubiquitinated p53.
